# Choosing the appropriate pharmacotherapy for nonspecific chronic low back pain

**DOI:** 10.1186/s13018-022-03426-5

**Published:** 2022-12-21

**Authors:** Filippo Migliorini, Nicola Maffulli

**Affiliations:** 1 Department of Orthopedic, Trauma, and Reconstructive Surgery, University Clinic Aachen, RWTH Aachen University Clinic, 52064 Aachen, Germany; 2 Department of Orthopaedic and Trauma Surgery, Eifelklinik St.Brigida, 52152 Simmerath, Germany; 3 Department of Medicine, Surgery and Dentistry, University of Salerno, 84081 Baronissi, SA Italy; 4 Queen Mary University of London, Barts and the London School of Medicine and Dentistry, Mile End Hospital, London, E1 4DG England; 5 School of Pharmacy and Bioengineering, Keele University Faculty of Medicine, Stoke On Trent, England

**Keywords:** Spine, Low back pain, Chronic, Pharmacological therapy

## Abstract

The pharmacological management of nonspecific chronic low back pain (NCLBP) aims to restore daily activities and improve the quality of life. No magic bullet exists for NCLBP; interventions to reduce pain and disability are available, but long-term results are unpredictable. Education in this regard needs to improve. This is often hard to accept for clinicians and patients, and provides a fertile soil to quacks, faith healers, and gurus to promote miraculous non-evidence-based solutions. The management of NCLBP is not well codified and extremely heterogeneous, and residual symptoms are common. Depending on the individual severity of NCLPB, pharmacological management may range from nonopioid to opioid analgesics. It is important to identify patients with generalized sensory hypersensitivity, who may benefit from a dedicated therapy. In this editorial, we provide an evidenced-based overview of the principles of pharmacological management of NCLPB.

Low back pain is the single most common cause of pain and disability in industrialised countries, with high burden in the health care systems worldwide. A rigorous diagnostic algorithm should be conducted to investigate the cause of pain and to initiate the appropriate management. Identification of possible red flags is mandatory in the assessment of low back pain. However, up to 95% of low back pain patients does not have an identifiable anatomical basis, and the pain is therefore defined as nonspecific [1]. Low back pain is considered “chronic” when symptoms last more than three months [2]. The pharmacological management of nonspecific chronic low back pain (NCLBP) aims to improve patient daily activities and quality of life. The management of NCLBP is not well codified and extremely heterogeneous, and residual symptoms are common. No magic bullet exists: interventions to reduce pain and disability are available, but long-term outcomes are unpredictable. Non-pharmacological methodologies are recommended as a first-line therapy for NCLBP. Among them, manipulations, appropriately and well-structured physical activity, education and psychological support are most commonly recommended. Pharmacological management should be considered as co-adjuvant to non-pharmacological therapy and should be guided by the symptoms reported by the patients. Clinicians have to choose from drugs with very modest effects and variable risk profiles. Hence, the widespread recommendations to use pharmacological options as a last resort. The benefits are just not there to justify the routine prolonged use of any given drug in NCLBP: this is a major challenge, and it is often hard to accept for clinicians and patients, providing a fertile soil for quacks, faith healers, and gurus to promote miraculous non-evidence-based solutions. Education in this regard needs to improve. Depending on the individual severity of NCLPB, pharmacological management may range from nonopioid to opioid analgesics. Evidence-based strategies to manage NSLPB are limited and heterogeneous. In the present editorial, the main pharmacotherapeutic options to manage patients with NCLPB are presented and critically discussed.

The little high level scientific evidence available advocates the stepwise administration of paracetamol (acetaminophen), non-steroidal anti-inflammatory drugs (NSAIDs), and opiates. Considerable uncertainty exists about the clinical efficacy of paracetamol for NCLBP. Recommendations for paracetamol are variable: some guidelines recommend it for the management of NCLBP, and others discourage its administration.

Short to midterm NSAIDs administration in NCLBP has been supported by high-quality evidence [3–5]. Most guidelines advocate the use of NSAIDs in NCLBP. However, their long-term efficacy in NCLBP is unclear and not supported by current evidence. Non-selective NSAIDs (e.g. ibuprofen, diclofenac) are contraindicated in patients with a history of gastrointestinal ulcers and bleeding, and daily protonic pomp inhibitors (e.g. pantoprazole, omeprazole) should be concurrently administered. On the other hand, the use of selective NSAIDs (e.g. celecoxib, etoricoxib) for longer than three months is contraindicated in patients with high risk of cardiovascular and renal diseases. Opioids should be combined with nonopioid pharmacotherapy and administered for the shortest possible period to promote improvement in pain and disability, with most placebo-controlled RCTs shorter than 6 weeks [3, 6, 7]. The current guidelines support weak opioids administration for the shortest period if other analgesics are ineffective, not tolerated, or contraindicated. Given their high risk of abuse, addiction, tolerance, and desensitisation, opioids administration should be cautiously monitored. Opioid therapy should be considered as a last resort, only to be implemented when the benefits are expected to outweigh risks, and pre-established treatment targets should be arranged with the patients. If targets are not reached, opioid therapy should be revisited. If dose increases do not provide sustained improvement, they should be reversed. Weak opioids (e.g. tramadol, tilidine/naloxone) with immediate-release at the lowest effective dosage should be used first, and strong opioids (e.g. oxymorphone, buprenorphine) should considered as a last resort [7]. Corticosteroids and antibiotics administration demonstrated no benefit in NCLBP and expose patients to additional risks. Duloxetine should be administered as second-line therapy in patients with features of generalised pain disorders, especially in those with multiple and chronic painful sites [8–10]. Selective serotonin–noradrenaline-reuptake-inhibitors (SSRI) and tricyclic antidepressants (TCA) should not be used on a regular basis and should be considered only if relevant psychiatric comorbidities coexist. The use of antidepressants in NCLBP is not supported by current guidelines. Current evidence on flupirtine, topiramate, and gabapentinoids administration for NCLBP is limited and demonstrates considerable risk of adverse effects without evident benefit, with high costs, and addiction risks [11–13]. Most guidelines worldwide do not recommend the use of flupirtine, topiramate, and gabapentinoids in NCLBP. The benefit of central myorelaxants (benzodiazepines, cyclobenzaprine) in NCLBP is uncertain. Current guidelines on myorelaxants in NCLBP are contradictory: some guidelines recommend their use as second-line therapy in exacerbations, and others advise against them. Non-benzodiazepine myorelaxants might promote minimal improvement in NCLBP at approximately two weeks in isolation or as co-medication. However, given their multiple collateral effects and interactions, addiction, and tolerance, along with the limited evidence, central myorelaxants should probably not be used outside the remits of clinical trials. Metamizole, also known as dipyrone, may be used in isolation or in association with NSAIDs in NCLBP. In less than one case pro million prescriptions, the use of metamizole is associated with agranulocytosis. Agranulocytosis occurs within the first weeks of metamizole use; however, a latency of up to several months may be possible, making it difficult to identify the association. Since the 1960s, metamizole has been withdraw from the market or was never approved in many countries. Metamizole in NCLBP has been poorly investigated, and no evidence-based indications can be inferred.

The management of NCLBP is not well codified and extremely heterogeneous, and residual symptoms are common. Guidelines and high-quality clinical trials on NCLBP have been published; however, there is no consensus concerning the optimal pharmacological approach and shared international guidelines are missing. The pharmacological management of NCLBP is not curative and is certainly not a substitute to non-pharmacological modalities. Pharmacological management should be considered in NCLBP exacerbations. Non-pharmacological strategies should be implemented constantly to maximise symptoms control. Manipulations, structured physical activity, education and psychological support are the most commonly recommended non-pharmacological methods to ensure symptoms control. In daily living, a correct posture, comfortable pillow and mattress, optimal nutrition and hydration, stress control also are associated with improved symptoms control.

## Data Availability

This study does not contain any third material.

## Abbreviations

NCLBP: Nonspecific chronic low back pain
NSAIDs: Non-steroidal anti-inflammatory drugs
